# Using Participatory Workshops to Assess Alignment or Tension in the Community for Minimally Invasive Tissue Sampling Prior to Start of Child Mortality Surveillance: Lessons From 5 Sites Across the CHAMPS Network

**DOI:** 10.1093/cid/ciz563

**Published:** 2019-10-09

**Authors:** John Blevins, Elizabeth O’Mara Sage, Ahoua Kone, Maria Maixenchs, Pratima L Raghunathan, Rui A Guilaze, Saquina Cossa, Zerihun Girma, Yosef Zegeye, Caroline Ackley, Faruqe Hussain, Saiful Islam, Nellie Myburgh, Noni Ngwenya, Shabir A Madhi, Peter Otieno, Kennedy Ochola, Khátia Munguambe, Robert F Breiman

**Affiliations:** 1 Emory Global Health Institute, Emory University, Atlanta, Georgia, USA; 2 Center for Global Health, Centers for Disease Control and Prevention, Atlanta, Georgia, USA; 3 ISGlobal, Hospital Clinic-Universitat de Barcelona, Spain; 4 Centro de Investigacao en Saude de Manhica (CISM), Maputo, Mozambique; 5 College of Health and Medical Sciences, Haramaya University, Harar, Ethiopia; 6 Department of Infectious Disease Epidemiology, London School of Hygiene and Tropical Medicine, United Kingdom; 7 PEI, Infectious Disease Division, icddr,b, Dhaka, Bangladesh; 8 University of New South Wales, Syndey, Australia; 9 Medical Research Council, Respiratory and Meningeal Pathogens Research Unit, University of the Witwatersrand, Faculty of Health Sciences Johannesburg, South Africa; 10 Department of Science and Technology/National Research Foundation, Vaccine Preventable Diseases, University of the Witwatersrand, Faculty of Health Sciences, Johannesburg, South Africa; 11 Kenya Medical Research Institute, Kisumu, Kenya; 12 Community Health Department, Eduardo Mondlane University, Faculty of Medicine, Maputo, Mozambique

## Abstract

The Child Health and Mortality Prevention Surveillance (CHAMPS) program is a 7-country network (as of December 2018) established by the Bill & Melinda Gates Foundation to identify the causes of death in children in communities with high rates of under-5 mortality. The program carries out both mortality and pregnancy surveillance, and mortality surveillance employs minimally invasive tissue sampling (MITS) to gather small samples of body fluids and tissue from the bodies of children who have died. While this method will lead to greater knowledge of the specific causes of childhood mortality, the procedure is in tension with cultural and religious norms in many of the countries where CHAMPS works—Bangladesh, Ethiopia, Kenya, Mali, Mozambique, Sierra Leone, and South Africa. Participatory Inquiry Into Community Knowledge of Child Health and Mortality Prevention (PICK-CHAMP) is a community entry activity designed to introduce CHAMPS to communities and gather initial perspectives on alignments and tensions between CHAMPS activities and community perceptions and priorities. Participants’ responses revealed medium levels of overall alignment in all sites (with the exception of South Africa, where alignment was high) and medium levels of tension (with the exception of Ethiopia, where tension was high). Alignment was high and tension was low for pregnancy surveillance across all sites, whereas Ethiopia reflected low alignment and high tension for MITS. Participants across all sites indicated that support for MITS was possible only if the procedure did not interfere with burial practices and rituals.

## Teaser

CHAMPS conducted community engagement workshops to assess alignments and tensions between community beliefs and practices and the minimally invasive tissue sampling procedure. The workshops revealed varying levels of tension across sites, with religious beliefs being a core driver of tension.

The Child Health and Mortality Prevention Surveillance (CHAMPS) program is a multiyear initiative to determine with greater specificity the causes of under-5 mortality in countries of the world with an under-5 mortality incidence of at least 50 per 1000 live births.

The CHAMPS program has been focused on mortality and demographic surveillance during the first 3-year phase (July 2015–June 2018), with plans to add pregnancy surveillance into site activities in the second 5-year phase. These activities have been carried out in population clusters of at least 100 000 people representing a variety of communities across the network including large cities, medium-sized towns, and rural villages. In-country partners (representing both governmental authorities and civil society organizations) can use surveillance data in subsequent phases to develop policies and programs to address the causes of under-5 mortality even as the surveillance activities continue. Multilateral partners and international donors plan to collaborate on these data-to-action initiatives beginning in the second phase (commencing in July 2018).

To determine the causes of under-5 mortality, CHAMPS sites gather body fluid and tissue samples from the bodies of children who have died, after obtaining parental permission. Mindful of low acceptance for complete diagnostic autopsies (CDAs) in many parts of the world, due in large part to religious and cultural beliefs [1–3], the CHAMPS program employs minimally invasive tissue sampling (MITS) that uses a special instrument with a spring-loaded needle mechanism to gather the samples without any incisions on the body [1, 4]. While some limited studies have demonstrated that community members are more supportive of MITS when compared to a CDA in some places where CDAs are not widely accepted [5, 6], research reveals that concerns about the procedure still remain [4]. Likewise, various studies indicate that social stigma from complications during pregnancy may lead to inaccurate reporting of such complications [7, 8], reinforcing the need to assess acceptability in regard to pregnancy surveillance. The CHAMPS program has devoted substantial resources to carry out formative research and support strong, sustained community engagement activities in all country sites due in large part to the numerous sensitive issues related to acceptability of the MITS procedure and of pregnancy surveillance.

The first community engagement activity sponsored by CHAMPS was a series of interactive workshops with community leaders and community members-at-large; these workshops were held in the local communities where surveillance would be implemented in each of the country sites. The workshops, entitled Participatory Inquiry Into Community Knowledge of Child Health and Mortality Prevention (PICK-CHAMP), have adapted an approach to community engagement known as participatory rural appraisal. The approach, widely employed in social and economic development initiatives, was developed by Robert Chambers and colleagues at the Institute for Development Studies at the University of Sussex, United Kingdom [9]. Participatory rural appraisal is grounded in the social theories of the Brazilian educator and philosopher Paulo Freire and reflects those theories in assuming that local communities are best equipped to identify their own priorities in relation to health and development initiatives, and that such initiatives can only be effective and sustainable when they align with the community’s interests [10]. While the CHAMPS program does not directly promote or provide health or psychosocial services in general (some CHAMPS sites offer limited clinical or psychosocial support to families in the catchment areas where the surveillance occurs), the program is nonetheless trying to build partnerships with community members, leaders, and organizations to identify common priorities and approaches to carrying out surveillance. To the extent feasible, CHAMPS seeks to identify, develop, and carry out sustainable, low-cost, shared activities to address the communities’ stated needs related to maternal and child health. All of these activities are well served by the processes employed in participatory rural appraisal that have been adapted for PICK-CHAMP workshops. For example, findings from PICK-CHAMP workshops in Kenya informed subsequent community health initiatives that offered nutritional counseling, antenatal screenings, and childhood immunizations. In Ethiopia, the CHAMPS team developed a curriculum on childhood health that included modules on nutrition, sanitation, and hygiene—topics named as most pressing in PICK-CHAMP workshops. This curriculum was subsequently implemented in local communities with community leaders, many of whom had participated in PICK-CHAMP, serving as co-trainers alongside local health workers.

**Figure 1.**
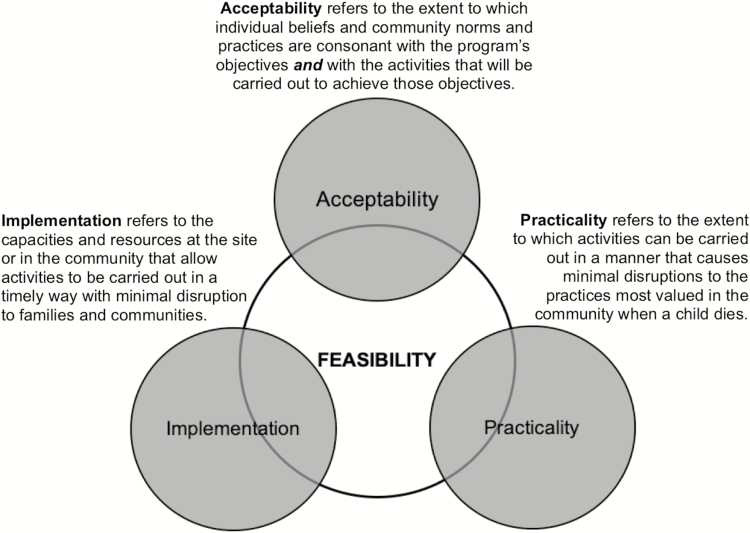
The conceptual framework for formative research to assess the feasibility of conducting mortality surveillance using minimally invasive tissue sampling across the Child Health and Mortality Prevention Surveillance network.

This article presents results from the workshop exercises to assess levels of alignment or tension between community priorities and CHAMPS activities and to identify the sources of tension so that qualitative research could be conducted to better understand those sources. Alignment and tension were defined in light of the conceptual framework for formative research developed by CHAMPS. This formative research, carried out by sociobehavioral science (SBS) teams in each site, assessed the feasibility of surveillance activities [11]. This was done through 3 primary constructs: acceptability, practicality, and implementation. In general, acceptability refers to levels of reaction, agreement, or disagreement with CHAMPS activities, especially MITS. Religious teachings and cultural beliefs, for example, affect acceptability of MITS when those teachings and beliefs represent a logic and moral framework at odds with the MITS procedure. Practicality refers to the extent to which surveillance activities conflict with community practices, timing, priorities, and/or resources related to death and burial. Understanding these conflicts allows for surveillance procedures to be developed that minimize disruption for the family and community. Again, using MITS as an example, practicality may refer to issues such as the time required to complete the procedure and the transport of the body because factors such as these will impact individuals’ willingness to consent to MITS, not on the basis of acceptability per se but on the basis of the level of disruption in relation to other activities that would take precedence over MITS. Finally, implementation generally refers to any issues associated with the requirements, resources, and/or logistics that must be addressed to carry out CHAMPS activities. For example, surveillance implementation may be delayed because training is necessary for facility staff or because the roles of government authorities in CHAMPS activities have not been established. In the analysis of the findings from PICK-CHAMP workshops, alignment was defined as any response that would support acceptability, practicality, and implementation, while tension was defined as any response that represented a barrier to acceptability, practicality, and implementation. Figure 1 represents the conceptual framework for assessing feasibility of CHAMPS activities by examining acceptability, practicality, and implementation.

While PICK-CHAMP workshops were conducted in all 7 CHAMPS sites, this article describes findings from 5 sites—Bangladesh, Ethiopia, Kenya, Mozambique, and South Africa (a description and further information on the sites can be found in a separate article by O’Mara et al in this supplement to *Clinical Infectious Diseases*—where activities were conducted subsequent to institutional review board (IRB) approval of SBS protocols [11].

## METHODS

**Design and Rationale**

PICK-CHAMP consists of 2 separate daylong workshops: one for community leaders contains 7 exercises, and one for community members-at-large contains 6 exercises. An annotated version of the PICK-CHAMP curriculum and various explanatory tables for measuring alignment and tension are included as Supplementary Data. The annotated curriculum in the Supplementary Data includes objectives and brief descriptions of all exercises for both workshops.

The rationale for designing the workshop content focused on answering the following 4 questions: (1) How do CHAMPS mortality activities align with or stand in tension with the community’s own priorities, norms, and perceptions? (2) What are the most powerful factors for community members that affect pregnancy and childhood health? (3) What are the most important activities that happen in the community when a woman discovers she is pregnant or when a child dies? (4) Who are the most influential individuals or organizations in the community with whom CHAMPS should work to build partnerships?

Participants’ responses in relation to the first question were used to frame community discussions and craft messages about CHAMPS that reflect the community’s point of view. Those responses also served as a reminder to CHAMPS staff that even when community members support CHAMPS, they will articulate the program’s benefits to the community from their own viewpoint and that viewpoint may not always reflect the benefits articulated by epidemiologists, research scientists, or clinical providers. Responses to the second question were used to assess the extent to which community perceptions of maternal and child health align with effective models of antenatal and pediatric care.

Responses in relation to the third question allowed CHAMPS to examine not only beliefs but also practices related to pregnancy and child death. This allowed the CHAMPS team to develop procedures for carrying out pregnancy and mortality surveillance in ways that minimize disruption to the parents, family, and community at these important and sensitive times. Responses in relation to the fourth question informed potential partnerships between CHAMPS and trusted community leaders or organizations. Participants in the community leaders’ workshops were asked about their own roles and responsibilities in support of maternal and child health and the ways that CHAMPS could support the leaders in fulfilling those roles.

### Implementation Approach

PICK-CHAMP workshops were the first activities carried out in the sites, with each site completing all workshops in a timeframe of 2–3 months. Participants for community leader workshops were drawn from among religious leaders, local political leaders (including representatives of minority parties), traditional leaders such as village chiefs (where applicable), traditional medical practitioners, maternal and child health providers, trusted elders, and leaders of local nongovernmental organizations. Participants for community member workshops were selected to ensure representation across ethnic groups, genders, religion, age, marital status, educational level, and socioeconomic status. Some sites are implementing CHAMPS in communities where they have carried out other programs; in such instances, the sites relied on existing social networks to advertise the workshops and recruit participants, supplementing those reached through those networks with invitations to others in order to achieve the representation among participants described above. For sites implementing CHAMPS in new communities where no earlier work had been carried out, staff first met with local leaders to introduce the program and solicit their support. Through such meetings, those leaders then introduced staff to individuals and organizations in the local community; participants for both workshops were identified through these processes using snowball sampling methods. The workshop curriculum was written in English and local CHAMPS staff in each country trained in SBS translated the content into the predominant language(s) of the local communities.

Subsequent to the workshops, CHAMPS staff entered participants’ responses into databases and analyzed the findings to inform formative research and the start of community engagement activities. In 6 of 7 CHAMPS sites, these activities preceded the initiation of mortality surveillance. The South Africa site was an exception, as it had previously piloted MITS and continued carrying out the procedure for deaths that occurred in a hospital facility as the site transitioned into the CHAMPS network.

### Data Collection

Demographic characteristics for each participant were gathered at registration. These characteristics included age, sex, marital status, religion, education, employment, and history of parenting (did the participant have any children; if so, what were their ages and had the participant experienced the death of any children). Participants were each assigned a unique identifying number and their responses were coded with that number so that responses could be analyzed by demographic variables without being tied to a participant’s name. In many instances, participants did not have the literacy level required to provide written responses to questions. This was the case in all community member workshops in every country; in these workshops participants offered verbal responses to questions raised in the exercises and CHAMPS staff members took notes of those responses, identifying the participant who offered a particular response through her or his unique identifier, which was printed on a nametag that participants wore. In some community leader workshops, participants wrote responses to questions on index cards that were labeled with their unique identifier. In other community leader workshops, verbal responses were recorded in the same way as in the community member workshops. Responses for each workshop were entered into a separate Access database file (Microsoft, Redmond, Washington) and each site provided the program office with those files. The responses were then exported into Excel files (Microsoft) for analysis.

### Analysis

Participants’ responses in each workshop were analyzed in light of the topics described above (see discussion on “Design and Rationale” in this section). In addition, responses for selected exercises were analyzed for alignment or tension with mortality or pregnancy surveillance (the “Alignment Tension Assessment Instrument” in the Supplementary Data notes how each exercise was analyzed). For community member workshops, the general alignment and tension score ranged from 0 to 4. For community leader workshops, the scores ranged from 0 to 8. Each score was combined, yielding a composite alignment score range from 0 to 12 and composite tension score of 0 to 12. Alignment and tension scores were also generated specifically for mortality surveillance and pregnancy surveillance. The range for the alignment and tension score for pregnancy surveillance was 0–12 and the range for MITS was 0–24.

The rationale behind the alignment/tension scoring is grounded in a number of assumptions informed by research in the field. Each of the exercises seeks to understand community perceptions related to pregnancy and childhood illness/death so that the acceptability of CHAMPS pregnancy and mortality surveillance activities as the first step of improving pregnancy and pediatric health outcomes could be assessed. Exercises 1–3 in both workshops assume that concepts of health and illness are generated in cultural contexts [12, 13]. While CHAMPS objectives are logical for practitioners and researchers coming from clinical and public health cultural frameworks informed by disciplines such as epidemiology and medicine, the cultural frameworks of local communities where CHAMPS is being implemented may not understand the purpose or logic of those same objectives or prioritize them in the same way. If participants could develop a number of different messages from their own framework that demonstrated the value or purpose of CHAMPS objectives, this would demonstrate an alignment with CHAMPS objectives. Similarly, if participants identified factors that impacted the course of pregnancy or child health that are readily addressed by clinical or public health programs, this would indicate an alignment between community perceptions and CHAMPS objectives.

Finally, alignment/tension scores for mortality surveillance carried more weight than those for pregnancy surveillance, and scores from community leaders carried more weight than those from community members. Greater weight was given to assessments of alignment/tension of mortality surveillance (including MITS) over pregnancy surveillance based on the literature that showed lower acceptance of autopsy procedures. Participants’ responses in the PICK-CHAMP workshops reinforced this finding; the levels of acceptability for mortality surveillance were far lower than those for pregnancy surveillance based on a number of different factors that are discussed below. This held true in every country. Finally, alignment/tension scores derived from community leaders’ responses were given greater weight than those derived from community members because of the greater extent of social relations and the greater number of internal and external ties of these leaders, each of which translated into greater social capital to influence community norms and perceptions [14, 15].

The Supplementary Data include the instrument for scoring alignment/tension. This instrument provides detailed information on the calculation of alignment/tension in the various exercises, the calculation of the alignment and tension scores for mortality and pregnancy surveillance, and the composite alignment/tension score for each site.

### Human Subjects Considerations

While the CHAMPS network currently consists of sites in 7 countries, findings from PICK-CHAMP workshops in 2 countries—Mali and Sierra Leone—are not included in this article because the activities were carried out there as stand-alone community engagement activities prior to ethical review and clearance of the broader SBS protocol. For the other 5 sites, PICK-CHAMP was included as part of SBS activities approved by institutional review boards in each country. In addition, the IRB at Emory University approved the SBS protocol that included PICK-CHAMP, and the US Centers for Disease Control and Prevention (CDC) Human Research Protection Office reviewed it and established reliance agreements with the IRBs at Emory and in each country where CDC staff is engaged [11].

## RESULTS

A total of 70 PICK-CHAMP workshops were conducted across the 5 sites, with a total of 1579 participants. The number of participants by country, along with their demographic characteristics, can be found in Table 1.

**Table 1.** Number of Workshops, Participants, and Participant Demographics by 5 Child Health and Mortality Prevention Surveillance Country Sites

Characteristic	Bangladesh: 14 Workshops		Ethiopia: 20 Workshops		Kenya: 8 Workshops		Mozambique: 14 Workshops		South Africa: 14 Workshops	
	CL^a^ (n = 173)	CM (n = 175)	CL (n = 225)	CM (n = 211)	CL (n = 96)	CM (n = 86)	CL (n = 156)	CM (n = 122)	CL (n = 67)	CM (n = 168)
Sex										
Male	106 (72)	87 (50)	92 (41)	160 (76)	59 (61)	33 (38)	78 (50)	37 (30)	62 (37)	53 (32)
Female	42 (28)	88 (50)	133 (59)	51 (24)	37 (39)	53 (62)	78 (50)	85 (70)	105 (63)	115 (68)
Marital status										
Never married/single	9 (6)	6 (3)	29 (13)	2 (1)	4 (4)	16 (19)	10 (6)	8 (7)	112 (67)	125 (74)
Monogamous marriage/ cohabitation	131 (89)	164 (94)	193 (86)	191 (91)	73 (76)	51 (59)	100 (64)	69 (57)	33 (20)	27 (16)
Polygamous marriage	0 (0)	0 (0)	0 (0)	0 (0)	13 (14)	10 (12)	0 (0)	0 (0)	0 (0)	0 (0)
Divorced	0 (0)	0 (0)	1 (<1)	4 (2)	0 (0)	2 (2)	10 (6)	5 (4)	14 (8)	9 (5)
Widowed	6 (4)	5 (3)	2 (1)	14 (7)	6 (6)	7 (8)	36 (23)	40 (33)	7 (4)	7 (4)
No response	2 (1)	0 (0)	0 (0)	0 (0)	0 (0)	0 (0)	0 (0)	0 (0)	1 (<1)	0 (0)
Education (highest level enrolled or completed)										
No school	3 (2)	18 (10)	32 (14)	82 (39)	2 (2)	2 (2)	56 (36)	48 (39)	2 (1)	1 (<1)
Primary	13 (9)	25 (14)	66 (29)	78 (37)	29 (30)	31 (36)	85 (55)	64 (52)	14 (8)	7 (4)
Secondary	65 (44)	62 (35)	29 (13)	33 (16)	39 (41)	42 (49)	12 (8)	10 (8)	125 (75)	127 (76)
Postsecondary	21 (14)	15 (9)	98 (44)	18 (9)	26 (27)	11 (13)	3 (2)	0 (0)	24 (14)	33 (20)
No response	46 (31)	55 (31)	0 (0)	0 (0)	0 (0)	0 (0)	0 (0)	0 (0)	2 (1)	0 (0)
Religion										
Christian	1 (1)	0 (0)	98 (44)	62 (29)	86 (90)	71 (83)	74 (47)	58 (45)	148 (89)	143 (85)
Hindu	47 (32)	51 (29)	0 (0)	0 (0)	0 (0)	0 (0)	0 (0)	0 (0)	2 (1)	0 (0)
Muslim	97 (66)	123 (70)	126 (56)	149 (71)	10 (10)	10 (12)	3 (2)	1 (1)	2 (1)	2 (1)
Traditional religion	0 (0)	0 (0)	0 (0)	0 (0)	0 (0)	5 (6)	64 (41)	44 (36)	6 (4)	10 (6)
Other	0 (0)	0 (0)	0 (0)	0 (0)	0 (0)	0 (0)	3 (2)	0 (0)	3 (2)	6 (4)
None	0 (0)	0 (0)	1 (<1)	0 (0)	0 (0)	0 (0)	3 (2)	3 (2)	1 (<1)	0 (0)
No response	3 (2)	1 (<1)	0 (0)	0 (0)	0 (0)	0 (0)	9 (6)	6 (5)	7 (4)	7 (4)
Death of child in past?										
Yes	31 (21)	74 (42)	55 (24)	73 (35)	44 (46)	36 (42)	115 (74)	86 (70)	49 (29)	32 (19)
No	117 (79)	101 (58)	170 (76)	138 (65)	52 (54)	50 (58)	41 (26)	36 (30)	118 (71)	136 (81)
Participant age, y										
Range	20–84	18–75	18/81	18–62	24–71	18–75	23–109	19–100	20–75	20–79
Mean/median	50/50	40/38	38/35	30/28	47/47	40/38	61/60	54/57	43/42	39/34

Data are presented as No. (%) unless otherwise indicated.

Abbreviations: CL, community leaders; CM, community members.

^a^Participants in one workshop of community leaders in Bangladesh (n = 25) did not complete the registration sheet that captured demographic information.

The PICK-CHAMP workshops revealed medium to high levels of overall alignment across the sites and medium to high levels of overall tension. Alignment was highest in South Africa, while tension was highest in Ethiopia. In addition to overall alignment/tension scores, alignment and tension specific for pregnancy and mortality surveillance activities was also calculated (Table 2). Alignment with pregnancy surveillance was high across all 5 sites, with low levels of tension. Levels of alignment with mortality surveillance were more varied. Mozambique showed the overall highest level of alignment and lowest level of tension; in contrast, Ethiopia showed a low level of alignment and a high level of tension (Table 2). For details on the calculations of these scores, see “Calculation for Alignment and Tension for CHAMPS Activities” in the Supplementary Data.

**Table 2.** Scores for Overall Alignment/Tension and Alignment/Tension for Pregnancy Surveillance, and Minimally Invasive Tissue Sampling Across 5 Child Health and Mortality Prevention Surveillance Country Sites

Site	Composite Score		Pregnancy Surveillance		MITS	
	Alignment	Tension	Alignment	Tension	Alignment	Tension
Bangladesh	9 (medium)	3 (medium)	12 (high)	1 (low)	21 (high)	3 (low)
Ethiopia	8 (medium)	6 (high)	12 (high)	1 (low)	11 (low)	13 (high)
Kenya	9 (medium)	4 (medium)	NA^a^	NA^a^	NA^a^	NA^a^
Mozambique	9 (medium)	3 (medium)	12 (high)	0 (low)	22 (high)	2 (low)
South Africa	10 (high)	4 (medium)	10 (high)	2 (low)	19 (high)	5 (low)

Abbreviations: MITS, minimally invasive tissue sampling; NA, not applicable.

^a^Scores for pregnancy surveillance and MITS alignment/tension were generated based on responses to exercises not carried out in the Kenya site.

The data for each site demonstrated limited variation in alignment and tension by demographic characteristics with the exception of religion. The variation was greater between the sites, especially in the sources of alignment and tension. For example, most sites aligned with CHAMPS objectives of reducing child death and activities around pregnancy surveillance, but more experienced tension around the MITS procedure or the ability of CHAMPS to address community priorities through mortality surveillance. Key findings on specific factors impacting alignment and tension are presented below.

A further analysis of the results reveals a number of issues that have implications for implementation of surveillance activities for the CHAMPS country sites. Those issues, discussed below, include perceptions that MITS is at odds with religious beliefs and practices, tensions between community priorities and CHAMPS activities, tensions between CHAMPS activities and activities that happen in the community during pregnancy and the death of a child, and discrepancies in alignment/tension within different communities located within the same catchment area of the country.

### Tensions Related to Perceptions That MITS Is at Odds With Religious Beliefs and Practices

The tension between mortality surveillance/MITS and religious beliefs and religious practices was revealed in exercise 6. In that exercise, participants were asked to indicate whether they agreed, disagreed, or were uncertain in regard to 20 statements dealing with maternal and child health/surveillance; 10 of those statements related specifically to mortality surveillance. The percentage of responses to all 10 statements helped determine each site’s overall alignment/tension score for mortality surveillance. Two of the 20 statements in that exercise specifically refer to religious beliefs and practices in the context of MITS. Participants’ responses to each of those 2 statements are listed in Table 3.

**Table 3.** Community Members’ Responses to Statements That Address Religious Beliefs and Practices in Relation to Mortality Surveillance Across 5 Child Health and Mortality Prevention Surveillance Country Sites

Statement	Agree	Disagree	Uncertain
*Medicine and science may help us care for a child’s body during life, but care for a child’s body at death is the work of faith and of God alone.*			
Bangladesh (n = 122)	121 (100)	0 (0)	1 (<1)
Hindu (n = 39 [32%])	38 (97)	0 (0)	1 (3)
Muslim (n = 83 [68%])	83 (100)	0 (0)	0 (0)
Ethiopia (n = 211)	47 (22)	163 (77)	1 (<1)
Christian (n = 64 [30%])	19 (30)	44 (69)	1 (1)
Muslim (n = 147 [70%])	28 (19)	119 (81)	0
Kenya (n = 86)	34 (41)	51 (58)	1 (1)
Christian (n = 71 [83%])	30 (42)	40 (56)	1 (1)
Muslim (n = 10 [12%])	3 (30)	7 (70)	0 (0)
Traditional beliefs (n = 5 [6%])	1 (20)	4 (80)	0 (0)
Mozambique (n = 117)	35 (30)	76 (65)	6 (5)
Christian (n = 56 [48%])	20 (36)	34 (61)	2 (4)
Muslim (n = 1 [1%])	0 (0)	1 (100)	0 (0)
Traditional beliefs (n = 51 [44%])	10 (20)	38 (75)	3 (6)
Not religious (n = 3 [3%])	1 (33)	1 (33)	1 (33)
No response (n = 6 [5%])	4 (67)	2 (33)	0 (0)
South Africa (n = 121)	68 (57)	27 (22)	26 (22)
African traditional religion (n = 7 [6%])	3 (43)	2 (29)	2 (29)
Christian (n = 101 [83%])	59 (58)	24 (24)	18 (18)
Muslim (n = 2 [2%])	1 (50)	1 (50)	0 (0)
Other (n = 5 [4%])	2 (40)	0 (0)	3 (60)
No response (n = 6 [5%])	3 (50)	0 (0)	3 (50)
All community member respondents (n = 657)	305 (46)	317 (48)	35 (3)
Christian (n = 292 [44%])	128 (44)	142 (48)	22 (8)
Hindu (n = 39 [6%])	38 (97)	0 (0)	1 (3)
Muslim (n = 243 [37%])	115 (47)	128 (53)	0 (0)
Traditional beliefs (n = 63 [10%])	14 (22)	44 (70)	5 (8)
Other (n = 5 [1%])	2 (40)	0 (0)	3 (60)
Not religious (n = 3 [<1%])	1 (33)	1 (33)	1 (33)
No response (n = 12 [2%])	7 (58)	2 (17)	3 (25)
*I would not agree to any kind of procedure if it meant that I could not bury my child according to my faith or tradition.*			
Bangladesh (n = 120)	91 (76)	28 (23)	1 (1)
Hindu (n = 38 [32%])	33 (87)	4 (11)	1 (3)
Muslim (n = 82 [68%])	58 (71)	24 (29)	0 (0)
Ethiopia (n = 211)	17 (8)	194 (92)	0 (0)
Christian (n = 64 [30%])	15 (23)	49 (77)	0 (0)
Muslim (147 [70%])	2 (1)	145 (99)	0 (0)
Kenya (n = 86)	19 (22)	64 (74)	3 (3)
Christian (n = 71 [83%])	13 (18)	55 (77)	3 (4)
Muslim (n = 10 [12%])	5 (50)	5 (50)	0 (0)
Traditional beliefs (n = 5 [6%])	1 (20)	4 (80)	0 (0)
Mozambique (n = 115)	60 (52)	55 (48)	0 (0)
Christian (n = 54 [47%])	30 (56)	24 (44)	0 (0)
Muslim (n = 1 [1%])	1 (100)	0 (0)	0 (0)
Traditional beliefs (n = 51 [44%])	26 (50)	25 (50)	0 (0)
No religion (n = 3 [3%])	1 (33)	2 (67)	0 (0)
No response (n = 6 [5%])	2 (33)	4 (67)	0 (0)
South Africa (n = 122)	77 (63)	30 (25)	15 (12)
African traditional religion (n = 7 [6%])	4 (57)	3 (43)	0 (0)
Christian (n = 103 [83%])	66 (64)	25 (24)	12 (11)
Muslim (n = 2 [2%])	1 (50)	0 (0)	1 (50)
Other (n = 5 [4%])	3 (60)	1 (20)	1 (20)
No response (n = 5 [4%])	3 (60)	1 (20)	1 (20)
All community member respondents (n = 654)	264 (40)	371 (57)	19 (3)
Christian (n = 292 [44%])	124 (42)	153 (52)	15 (5)
Hindu (n = 38 [6%])	33 (87)	4 (11)	1 (3)
Muslim (n = 242 [37%])	67 (28)	174 (72)	1 (<1)
Traditional beliefs (n = 63 [10%])	31 (49)	32 (51)	0 (0)
Other (n = 5 [1%])	3 (60)	1 (20)	1 (20)
Not religious (n = 3 [<1%])	1 (33)	2 (67)	0 (0)
No response (n = 11 [2%])	5 (45)	5 (45)	1 (9)

Data are presented as No. (%) unless otherwise indicated.

In both statements, an “agree” response was interpreted to indicate some level of tension with mortality surveillance and MITS. There was no common pattern to which religious tradition was correlated with participant responses that indicated tension. In Bangladesh, for example, a high percentage of Muslim participants provided responses to each statement that were in tensions with mortality surveillance (100% of responses for statement 9 and 71% of responses for statement 10); conversely, in Ethiopia 19% of Muslim participants offered a response to statement 9 in tension with mortality surveillance and only 1% of Muslim participants offered such a response to statement 10. Responses by Christian participants in South Africa to statement 9 indicated tension at a rate (58%) almost twice that of responses by Christian participants in Ethiopia (30%); the discrepancy was even greater for statement 10, with tension at a rate of 64% in South Africa and 23% in Ethiopia. These kinds of findings reinforce findings in anthropological and religious studies which demonstrate that religion is not monolithic but highly variable across cultural contexts and challenge any assumption that religious objections to health initiatives could be addressed with a generalized set of messages that referenced either the doctrines or sacred texts of a specific religion [16].

**Figure 2.**
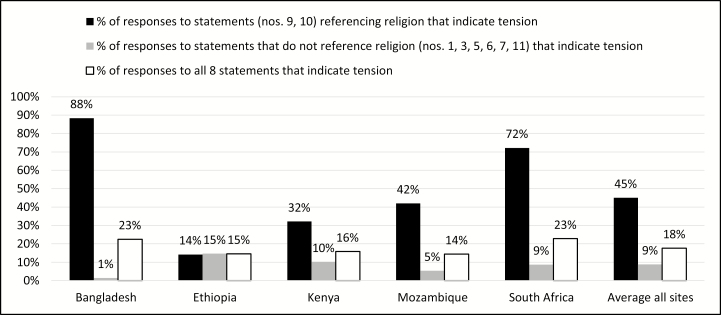
Percentage of responses to 8 statements referencing mortality surveillance that indicate tension.

By themselves, these statements do not demonstrate that communities are irrevocably opposed to MITS (in fact, overall alignment with mortality surveillance was high in Bangladesh even though tension related to both of these statements was also high); rather, they serve as a reminder that many community members will not prioritize CHAMPS activities over religious beliefs/practices at the time of death. Therefore, to successfully implement CHAMPS in these communities, CHAMPS activities must not be seen as contrary to religious beliefs or interfere with such practices. As sites began to implement MITS, this was addressed in concrete ways by ensuring that the time for conducting MITS was minimized so as not to delay burial, and the distance the body was transported was minimized because of religious beliefs among some that transport represented an unnecessary action that delayed burial. A total of 8 statements specifically asked about mortality surveillance in the context of community priorities. Statements 9 and 10 (Table 3) examined the context of religion; the 6 others examined other topics. With the exception of Ethiopia, responses that indicated tension were far more frequent in the 2 statements that referenced religion in comparison to the 6 statements that do not refer to religion (the percentage of responses indicating tension were basically the same in Ethiopia regardless of the issue referenced). See Figure 2 for the percentages of statements indicating tension.

### Tensions With CHAMPS Activities Addressing Community Priorities

In exercise 4 of the community leaders workshops, participants were asked to name the ways that CHAMPS could support them in their efforts to improve maternal and child health in their communities. Participants’ responses were coded as “feasible” (CHAMPS could directly support such efforts at the present time), “not feasible” (the suggestion was beyond the scope and capacity of CHAMPS and would remain so over the duration of the program), or “other” (CHAMPS could not support such efforts at the present time but could possibly offer support in the future through partnerships that would be built over the course of the project). In Ethiopia, only 32% of the suggestions made by participants were actually feasible and in Mozambique, 48% were feasible. These scores indicate a lower alignment with CHAMPS objective and activities, as well as high expectations or needs in the community that CHAMPS cannot address. The percentages of feasible activities in the other 3 sites were higher: 57% in Bangladesh, 64% in Kenya, and 66% in South Africa.

### Tension Between CHAMPS Surveillance and Important Community Activities During Pregnancy and During the Death of a Child

For all sites, the majority of activities surrounding pregnancy were not in tension with CHAMPS activities. In exercise 5 of the community leaders’ workshops, participants were asked to name the things that occur in the community when a woman finds out she is pregnant; they were then asked to identify whether pregnancy surveillance would conflict with carrying out those activities. In 3 sites, the percentage of activities that conflicted was <5%; South Africa had the highest percentage of activities that conflicted with pregnancy surveillance, at 26%. Conflict in relation to mortality surveillance was higher in some sites. Participants were asked to identify the things that occur in the community when a child dies and then asked to identify whether mortality surveillance (including MITS) would conflict with carrying out these activities. While conflict in Bangladesh and Mozambique was quite low (at 3% in each country), the percentage of activities that conflicted with mortality surveillance in South Africa was 29% and in Ethiopia, the percentage was 60%.

There was also tension related to beliefs about the acceptability of the MITS procedure in some sites. In exercise 6 of the community members’ workshop, participants were asked to provide their opinion on the claim that removing tissue samples from a deceased child was wrong even if it helps to determine cause of death (exercise 6, statement 1). Twenty-three percent of community members in South Africa agreed that such a procedure was wrong. In the Kenyan and Mozambican sites, approximately 10% of community members expressed this belief. Another statement in the same exercise also demonstrated tensions related to mortality surveillance from other factors. Twenty-one percent of all participants expressed a general belief that no parent should ever consent to tissue removal after a child’s death. This level of disagreement among some members in the community might indicate that parents who consent would be stigmatized in some local communities by at least some of their neighbors. This number was highest in Ethiopia, with 38% of participants expressing this belief.

### Discrepancies Across Communities Within the Same Surveillance Site

While the percentages cited above reflect the aggregate findings across all the PICK-CHAMP workshops conducted in that particular country site, there were instances of great discrepancies across different communities within the same site where workshops were conducted. This was most pronounced in South Africa. An analysis of participants’ responses to the following 5 statements addressing mortality surveillance in exercise 6 of the community members’ workshop demonstrates a broad range of responses:

### Tensions With Mortality Surveillance

- Statement 1: It is wrong to remove small samples of tissue from a child after she has died even if tests done on that tissue could tell you how she died.
- Statement 9: Medicine and science may help us care for a child’s body during life but care for a child’s body at death is the work of faith and of God alone.
- Statement 10: I would not agree to any kind of procedure if it meant that I could not bury my child according to my faith or tradition.

### Tensions With Pregnancy Surveillance

- Statement 14: While medical care for pregnant mothers and children is important, it is not easy for them to get the care they need.

### Community Tensions With Research

- Statement 19: Researchers don’t care about our community, but only about the information they collect.

**Figure 3.**
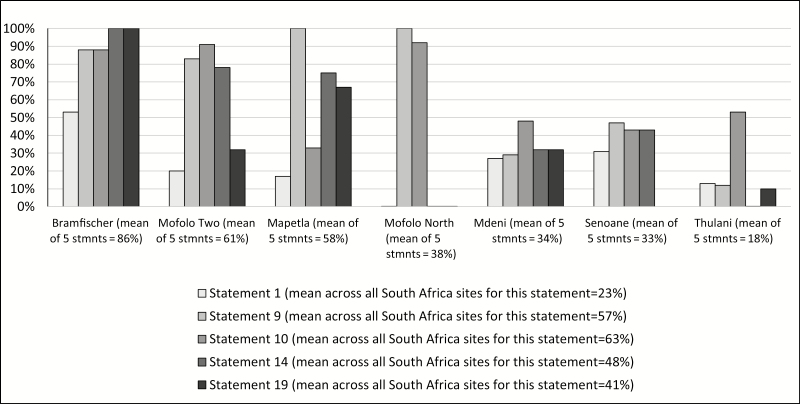
Percentage of participants’ responses to selected statements indicating tension with Child Health and Mortality Prevention Surveillance in South Africa. Abbreviation: stmnts, statements.

In one site (Bramfischer), 86% of participant responses indicated tension with mortality surveillance, whereas only 18% of responses in Thulani indicated tension. Further, in some of the clusters within the South Africa catchment, participants’ responses were widely divergent with responses to some statements indicating tension while responses within the same site to other statements indicated alignment. Figure 3 shows the range of responses in each of the clusters across the catchment areas in South Africa.

The disparities across the communities in South Africa where PICK-CHAMP was carried out is even more striking when one considers that these communities comprise the CHAMPS surveillance site in the country and comprise a small geographic area of neighborhoods within the same urban subdistrict of Johannesburg.

### Application of PICK-CHAMP Findings to Other CHAMPS Activities

PICK-CHAMP workshop findings on alignment and tension provided insight into the factors related to both acceptability (eg, in assessing participants’ responses to the following: “It is wrong to remove small samples of tissue from a child after she has died even if tests done on that tissue could tell you how she died”) and practicality (eg, in assessing participants’ responses to the following: “I would not agree to any kind of procedure if it meant that I could not bury my child according to my faith or tradition”). Understanding the drivers of acceptability and practicality informs the resources and logistics issues that must be addressed in relation to implementation.

PICK-CHAMP has also informed subsequent community engagement activities. PICK-CHAMPS findings have been used to inform approaches to 3 community engagement objectives: (1) identify and create partnerships with trusted individuals and organizations working on maternal and child health; (2) working with these partners, develop and sponsor feasible community activities in support of maternal and child health; and (3) demonstrate respect and appreciation for families who participate in CHAMPS activities, and for all children in the community through culturally appropriate activities and symbols. In exercise 6 in the community leaders’ workshop and exercise 7 in the community members’ workshop, participants named trusted organizations and individuals with whom CHAMPS could partner. CHAMPS SBS teams subsequently followed up with those who were named and established partnerships as feasible. In exercise 4 in the community leaders’ workshop, participants listed the ways that CHAMPS could support them as they carried out their roles and responsibilities in support of maternal and child health. Each of these responses was coded as feasible (CHAMPS could readily support such efforts), unfeasible (such efforts were beyond the capacity of CHAMPS to support), or other (supporting such efforts was not currently feasible but might be through partnership with local community networks). Those efforts labeled as “other” were prioritized in developing partnerships and the subsequent maternal and child health activities that CHAMPS is sponsoring or supporting.

Finally, the PICK-CHAMP findings that address religious and cultural norms helped inform the kinds of activities that could appropriately convey messages of respect and appreciation to families and communities. For example, some CHAMPS sites have an imam, pastor, or priest from the community available as part of the MITS team if parents request the religious leader’s involvement. In addition, one site also provides burial clothes for the child’s body after the procedure is completed. These acts convey 2 messages: (1) they demonstrate that the program respects the families’ religious beliefs and practices, and (2) they address critical issues of timing of the MITS procedure in a way that minimizes disruption to religious and community rituals.

Assessing disparities in alignment/tension across different communities within the same country allows CHAMPS SBS teams to prioritize community engagement activities in certain high-tension areas or to tailor the types of activities in certain communities to address suspected sources of tension based on PICK-CHAMP findings. For example, multiple initiatives are under way in Ethiopia, the country with the highest reported tension. Efforts have been made to establish a community advisory board consisting of key stakeholders. Community leaders, including religious leaders, teachers, and health professionals, attended a training of trainers initiative to provide health education in local communities; the initiative included modules on CHAMPS objectives and activities as well as feasible approaches for improved child health by addressing nutrition and water, sanitation, and hygiene. These trainers are now carrying out workshops on these topics alongside local health professionals to build trust with the community. Finally, the site is deploying “theatre for development,” a well-established method for community-based participatory action, to engage communities in new platforms to discuss CHAMPS activities, especially MITS.

## CONCLUSIONS

CHAMPS staff across disciplines have utilized PICK-CHAMP findings as they developed protocols and standard operating procedures related to consent, death notification, and family follow-up. Those findings have also informed planning related to MITS procedures to address concerns related to the timing of the procedure and the transport of the body in light of religious practices.

In summary, communities strongly align with CHAMPS objectives about identifying the causes of death in children and the causes of pregnancy complications in expectant mothers. Alignment remains high in regard to the specific activities CHAMPS will carry out as part of its pregnancy surveillance efforts. However, we observed lower alignment across the CHAMPS sites in regard to the activities that comprise mortality surveillance, especially the MITS procedure. The sources of tension related to mortality surveillance are varied and impact alignment/tension to varying degrees across the sites. In general, the factor of religious beliefs/practices generates the highest levels of tension with mortality surveillance. This might be because the systematic use of MITS for postmortem diagnostic testing and cause of death assignment in many childhood deaths is fairly new, even though postmortem needle biopsies have been carried out in a small number of cases for many years [17]. Religious leaders in the communities where CHAMPS is working require time to understand the procedure and how it relates to their religious beliefs and practices. The social authority of religious leaders has practice implications in some CHAMPS sites working in community contexts that are predominantly Muslim—specifically, the need to secure a fatwa from a local council of imams to indicate the religious permissibility of the MITS procedure.

PICK-CHAMP findings—most notably the calculation of alignment/tension scores and the coding of the sources of alignment/tension—have proven useful for prioritizing subsequent CHAMPS activities in formative research and community engagement for the SBS teams and for informing protocols and standard operating procedures for conducting MITS and mortality surveillance. PICK-CHAMP workshops elicited the input of community members-at-large and community leaders as part of the very first activities undertaken, employing established approaches for community engagement. In employing PICK-CHAMP, the CHAMPS network has demonstrated a commitment to the local communities where we are working. By establishing partnerships with organizations and trusted leaders, CHAMPS teams are working to demonstrate their commitment to improving overall maternal and child health over time and not only to generating and extracting epidemiological data through surveillance activities.

## Supplementary Data

Supplementary materials are available at *Clinical Infectious Diseases* online. Consisting of data provided by the authors to benefit the reader, the posted materials are not copy-edited and are the sole responsibility of the authors, so questions or comments should be addressed to the corresponding author.

## Notes

***Financial support.*** This work was supported by the Bill & Melinda Gates Foundation [OPP1126780].

***Supplement sponsorship.*** This supplement is sponsored by the Emory Global Health Institute and the Bill & Melinda Gates Foundation.

***Potential conflicts of interest.*** S. A. M. has received grants from the Bill & Melinda Gates Foundation, Pfizer, Glaxo Smith Kline, Biovac, Novavax, and Sanofi Pasteur; and honoraria from The Bill & Melinda Gates Foundation. All other authors report no potential conflicts of interest. All authors have submitted the ICMJE Form for Disclosure of Potential Conflicts of Interest. Conflicts that the editors consider relevant to the content of the manuscript have been disclosed.
